# A Retrospective Audit of Statin Prescription Practices in Diabetic Patients With Cardiovascular Risk

**DOI:** 10.7759/cureus.90152

**Published:** 2025-08-15

**Authors:** Muhammad Adnan Khaliq, Sabih Nofal, Samia Israr, Ali Husnain, Muhammad Fakhar Hayat, Muhammad Zarrar Arif Butt, Saadia Kanwal

**Affiliations:** 1 Internal Medicine, Services Hospital, Lahore, PAK; 2 Vascular Surgery, Shalamar Hospital, Lahore, PAK; 3 Internal Medicine, District Head Quarter Hospital, Okara, PAK; 4 Cardiology, Shalamar Hospital, Lahore, PAK; 5 Cardiology, Fatima Memorial Hospital College of Medicine and Dentistry, Lahore, PAK; 6 General Surgery, Shalamar Hospital, Lahore, PAK

**Keywords:** atherosclerosis, audit, cardiovascular, diabetic, statin

## Abstract

Background

Diabetic patients are at increased risk of cardiovascular disease (CVD), and clinical guidelines recommend statin therapy for most individuals with diabetes aged ≥40 years or those with established atherosclerotic CVD (ASCVD).

Objective

The main objective of this audit was to evaluate the adherence to guideline-based statin prescribing practices among diabetic patients with cardiovascular risk and to identify gaps in prescription intensity and eligibility.

Materials and methods

A retrospective clinical audit was conducted on 131 diabetic patients at a tertiary care center. Data were collected retrospectively through a structured audit tool using patient medical records, including demographics (age, sex), duration of diabetes, comorbidities, lipid profile reports, cardiovascular history, and current statin prescription status (type, dose, and intensity).

Results

Out of 131 diabetic patients, 109 (83.2%) were eligible for statin therapy. Among them, 84 (77.1%) received any statin, while 25 (22.9%) remained untreated despite eligibility. High-intensity statins were prescribed in only 28 (33.3%) cases. Among patients with ASCVD (n = 34), 85.3% were on statins, but only 55.9% received high-intensity therapy. In patients without ASCVD but with risk factors (n = 75), 73.3% received statins, mostly moderate-intensity. Statin use was significantly higher in males (83.8%) than in females (69.2%) (p = 0.041), and patients aged ≥50 were more likely to receive statins (p = 0.018).

Conclusions

This audit reveals underutilization and suboptimal dosing of statins in diabetic patients, especially among women and younger individuals. Strengthening clinical adherence through guideline dissemination, audit-feedback cycles, and electronic decision support systems may improve cardiovascular outcomes in this high-risk group.

## Introduction

The primary cause of morbidity and death in patients with diabetes mellitus is cardiovascular disease (CVD), which, among diabetic persons, is two to four times more prevalent than in non-diabetic groups concerning developing coronary artery disease, stroke, and peripheral vascular diseases [1]. The synergetic effect of chronic hyperglycemia and insulin resistance, dyslipidemia, endothelial dysfunction, and systemic inflammation is implied as being the cause of such an increased risk [2]. Considering this increased risk, all significant international guidelines, such as those of the American Diabetes Association (ADA), the American College of Cardiology (ACC), and the European Society of Cardiology (ESC), among others, advocate strongly for including statins in the center of preventive therapy in diabetics with established or high cardiovascular risk [3,4].

HMG-CoA reductase inhibitors, otherwise known as statins, efficiently lower the levels of low-density lipoprotein cholesterol (LDL-C) and have pleiotropic effects that include stabilization of the plaque, enhanced endothelial functions, and decreased vascular inflammation. The efficacy of statins in preventing cardiovascular events and mortality in diabetic patients, either in primary or secondary prevention, has been proven by many large-scale randomized controlled trials, such as the CARDS, HPS, and ASCOT-LLA experiments [5]. Clinical guidelines, therefore, recommend moderate-to-high-dose statins in all diabetic adults aged 40 years and above with other cardiovascular risk factors, independent of the initial levels of LDL-C [6].

Even within the unmet need, there is still a high discrepancy between guideline-based therapy and clinical practice in the real world, even with strong evidence and literature recommendations. Various reports conducted in different healthcare systems have found an undersupply of statins, according to the eligibility of diabetic patients, or wrongful prescribing of statins leading to wrong dosing [7]. Among them are clinical inertia, side effect apprehension, physician unfamiliarity with changing guidelines, patient non-adherence, and system-related factors like cost or absence of availability to lipid monitoring [8]. This evidence-to-practice gap is especially acute when dealing with patients with type 2 diabetes because of the increased number of comorbidities such as hypertension, obesity, and chronic kidney disease, which, in turn, complicate treatment algorithms as well as raise cardiovascular risk [9]. Besides, the under-recognition of the necessity of statin therapy may occur due to the diabetic dyslipidemia with high triglycerides, insufficiently high HDL-C, and relatively normal or slightly increased LDL-C because of the possibility that clinicians may pay attention only to the level of LDL-C without evaluating the atherogenic lipid profile.

Moreover, age and sex differences in the prescription of statins have been observed, with older people and females being less likely to receive statin therapy, despite very similar or higher absolute risk of cardiovascular events. Specialty, experience, and comfort using the guidelines are physician-related factors that have other impacts on the pattern of statin prescriptions [10,11]. In low-resource environments, especially in low- and middle-income countries (LMICs), these issues are usually exacerbated by the existence of inequality in healthcare infrastructure, uneven prescription habits, lack of national treatment guidelines, and poor health literacy on the part of the patients. Long-term use of statins, unavailability of administration programs to screen all individuals with dyslipidemia, and follow-ups further compromise optimal lipid treatment. LMIC studies have shown reduced statin use rates relative to high-income countries with high heterogeneity in levels of statin use amongst the publicly and privately funded sectors [12,13].

The primary goal of this audit is to assess prescribing practices of statins in diabetic patients with cardiovascular risk and their adherence to one of the internationally recognized clinical guidelines, e.g., ADA and ACC. The envisaged audit aims to identify the percentage of eligible patients who are initiated and receive appropriate statin treatment, assess the intensity of statin prescriptions concerning patient risk stratification, and identify trends of under-prescription or practices that deviate from the recommendations.

## Materials and methods

This clinical audit was conducted at Shalamar Hospital, Lahore, Pakistan, from March 2024 to March 2025. A total of 131 patients with a documented diagnosis of type 2 diabetes mellitus were included in the audit using non-probability consecutive sampling. Eligibility was determined based on clinical records of patients aged ≥40 years with at least one additional cardiovascular risk factor, such as hypertension, smoking, obesity, chronic kidney disease, or a history of cardiovascular events. Patients with incomplete medical records or contraindications to statin therapy (such as liver disease or statin intolerance) were excluded from the audit.

Data were collected retrospectively through a structured audit tool using patient medical records, including demographics (age, sex), duration of diabetes, comorbidities, lipid profile reports, cardiovascular history, and current statin prescription status (type, dose, and intensity). Prescription practices were evaluated against the 2022 ADA and ACC/AHA guideline recommendations for statin therapy in diabetic patients. Statin intensity was categorized as high, moderate, or low based on established definitions. Patients were then classified as appropriately treated or undertreated according to their cardiovascular risk profile. Data were compiled and analyzed using SPSS Statistics version 24 (IBM Corp. Released 2016. IBM SPSS Statistics for Windows, Version 24.0. Armonk, NY: IBM Corp.). Descriptive statistics were calculated for demographic and clinical variables, with results presented as frequencies, percentages, means, and standard deviations. The proportion of patients meeting guideline-recommended statin therapy was calculated, and stratified analyses were performed to explore associations between prescribing patterns and patient-related factors such as age, sex, and comorbid conditions. The findings were then compared to audit standards derived from current clinical guidelines to identify gaps in practice and potential areas for quality improvement.

## Results

Among 131 participants, the mean age was 58.7 ± 9.6 years, with 74 males (56.5%) and 57 females (43.5%). Hypertension was present in 97 patients (74.0%), obesity in 51 (38.9%), and 34 (25.9%) had a history of ASCVD. A total of 109 patients (83.2%) were eligible for statins, but only 84 (77.1%) of them were prescribed therapy. Among those treated, 28 (33.3%) received high-intensity, 48 (57.1%) moderate-intensity, and 8 (9.5%) low-intensity statins. The overall guideline adherence rate was 64.1% (Table 1).

**Table 1:** Baseline characteristics and statin prescription patterns of study participants (n = 131) Data are represented as mean ± SD or percentages (%). ASCVD: atherosclerotic cardiovascular disease

Variable	Value
Mean age (years)	58.7 ± 9.6
Gender – male	74 (56.5%)
Gender – female	57 (43.5%)
Hypertension	97 (74.0%)
Obesity	51 (38.9%)
History of ASCVD	34 (25.9%)
Patients aged ≥40 years	119 (90.8%)
Eligible for statin therapy	109 (83.2%)
Prescribed any statin	84 (77.1% of eligible)
Not prescribed statin despite eligibility	25 (22.9% of eligible)
High-intensity statin	28 (33.3% of prescribed)
Moderate-intensity statin	48 (57.1% of prescribed)
Low-intensity statin	8 (9.5% of prescribed)
Mean statin prescription rate (guideline adherence)	64.10%

Statin prescription was significantly associated with male gender (χ² = 4.17, p = 0.041) and age ≥50 years (χ² = 5.64, p = 0.018). Hypertension, obesity, and a history of ASCVD were not significantly associated with prescription rates (p > 0.05). Among eligible patients, 52 out of 62 males received statins versus 32 out of 47 females. Patients under 50 were less likely to be prescribed statins, with only 18 out of 29 receiving therapy (Table 2).

**Table 2:** Association between patient characteristics and statin prescription (n = 109 eligible patients) Significance level: p < 0.05. ASCVD: atherosclerotic cardiovascular disease

Variable	Category	Prescribed statin (n = 84)	Not prescribed (n = 25)	χ² (Chi-square)	p-value
Gender	Male	52	10	4.17	0.041*
	Female	32	15		
Hypertension	Present	69	18	0.73	0.392
	Absent	15	7		
Obesity	Present	34	11	0.12	0.729
	Absent	50	14		
History of ASCVD	Present	29	5	2.37	0.122
	Absent	55	20		
Age group	<50 years	18	11	5.64	0.018*
	≥50 years	66	14		

Patients on high-intensity statins had a slightly higher mean age (60.3 ± 8.1 years) compared to those on moderate- or low-intensity statins (57.4 ± 9.8 years), but the difference was not statistically significant (p = 0.211). Similarly, the mean age of those prescribed any statin (58.9 ± 9.4) versus those not prescribed (57.9 ± 10.1) also showed no significant difference (p = 0.301), indicating age was not a strong predictor of intensity or likelihood of statin use (Table 3).

**Table 3:** Comparison of mean age and statin intensity An independent t-test was used. SD: standard deviation

Group	Mean age (years)	SD	n	p-value
High-intensity statin users	60.3	8.1	28	0.211
Moderate/low-intensity users	57.4	9.8	56	
Prescribed statins (any)	58.9	9.4	84	0.301
Not prescribed statins	57.9	10.1	25	

## Discussion

This clinical audit was conducted to evaluate statin prescribing patterns among diabetic patients with cardiovascular risk and assess alignment with established clinical guidelines, particularly those of the ADA and ACC/AHA. The findings highlight a persistent evidence-practice gap, particularly in the underuse of high-intensity statins among eligible patients. Out of 131 diabetic patients reviewed, 109 (83.2%) met the eligibility criteria for statin therapy based on age and comorbid cardiovascular risk factors. However, only 84 (77.1%) of these eligible patients were prescribed any statin, and a mere 33.3% received high-intensity therapy. This reflects considerable underutilization, especially considering that high-intensity statins are the recommended standard for patients with known ASCVD or multiple risk factors.

These findings are consistent with previously published data. For example, Pokharel et al. evaluated real-world statin use in patients with diabetes and found that only 52% of eligible individuals received statin therapy, and a much smaller fraction received high-intensity statins as recommended [11]. Salami et al. similarly reported suboptimal adherence to guideline-directed statin therapy, with disparities in both access and prescribing patterns across demographics in the U.S. population [14]. Our audit showed an overall guideline adherence rate of 64.1% when both prescription and intensity were considered, which is moderately higher than in several international studies. A study by Elnaem et al. demonstrated that while statins were prescribed in nearly 70% of diabetic patients, high-intensity statin use in secondary prevention remained low, mirroring our findings [15]. A study in Sri Lanka noted that only 29% of high-risk diabetic patients received appropriate statin therapy as per guideline recommendations [16].

A focused sub-analysis in our audit found that patients with ASCVD (n = 34) had relatively higher adherence, with 85.3% prescribed statins. However, even in this group, only 55.9% were on high-intensity regimens, suggesting room for improvement even in well-established secondary prevention indications. Among patients with cardiovascular risk factors but no documented ASCVD (n = 75), 73.3% were prescribed statins, largely of moderate intensity. This implies possible therapeutic inertia, suboptimal risk assessment, or cautious prescribing tendencies. Another critical finding was the presence of gender disparities in statin prescription. Males were significantly more likely to be prescribed statins than females (83.8% vs. 69.2%, p = 0.041). This finding aligns with the previous research reports, which present lower statin use in women despite similar or higher cardiovascular risk [17,18]. Factors contributing to this disparity may include lower perceived cardiovascular risk in females, physician bias, or inadequate awareness of sex-specific risk factors. Similarly, age disparities were observed. Patients under 50 years of age were significantly less likely to be prescribed statins compared to those aged ≥50 years (p = 0.018), even when eligible. This mirrors findings from a previous study that emphasized the elevated lifetime cardiovascular risk associated with early-onset type 2 diabetes, underscoring the need for early preventive intervention [19].

The underlying reasons for such prescription gaps are likely multifactorial. These include clinician-related factors such as limited familiarity with evolving guidelines, concerns over adverse effects (e.g., myopathy, liver enzyme elevation), absence of up-to-date lipid panels, and lack of confidence in risk calculators. Systemic issues such as time constraints during consultations, lack of integrated decision-support tools, and variable access to medications in low-resource settings may also contribute. Studies from LMICs, including Pakistan, have reported similar challenges. A review by Bowry et al. highlighted infrastructure gaps, inconsistent national guidelines, and limited clinician training as major contributors to poor cardiovascular preventive care in LMICs [20]. To bridge the evidence-practice gap, strategies such as integrating guideline-based prompts into electronic health records, providing CME on ADA and ACC/AHA guidelines, and implementing structured audit-feedback loops are recommended. Enhancing access to lipid profile testing and incorporating risk stratification tools in outpatient care can further improve statin prescribing practices in diabetic patients.

While this audit offers valuable insights, it is limited by its retrospective design, selection bias, and single-center design, which may affect generalizability. Some records lacked lipid profile documentation, which could hinder accurate risk stratification. Additionally, the reasons for non-prescription, such as documented statin intolerance, patient refusal, or prior adverse reactions, were not consistently recorded, preventing a deeper understanding of clinical decision-making patterns.

## Conclusions

This clinical audit highlights a significant gap between guideline recommendations and actual prescribing practices of statins among diabetic patients at cardiovascular risk. Although a majority of patients were eligible for statin therapy, a considerable proportion were either not prescribed statins or were prescribed suboptimal intensity, particularly among females and younger patients. The findings underscore the need for improved adherence to clinical guidelines, enhanced physician awareness, and system-level strategies such as regular audits, clinical reminders, and decision-support tools.
